# Cecal epiploic appendage torsion in children and its diagnostic difficulties: a case report and review of literature

**DOI:** 10.1093/jscr/rjag033

**Published:** 2026-01-31

**Authors:** Isber Ademaj, Fisnik Kurshumliu, Naser Gjonbalaj, Arjanita Ademaj

**Affiliations:** Department of Pediatric Surgery, University Clinical Centre of Kosovo, Lagja e Spitalit PN, Prishtina 10000, Kosovo; Department of Pathology, University Clinical Centre of Kosovo, Lagja e Spitalit PN, Prishtina 10000, Kosovo; Department of Radiology, University Clinical Centre of Kosovo, Lagja e Spitalit PN, Prishtina 10000, Kosovo; Faculty of Medicine, University of Prishtina, Lagja e Spitalit PN, Prishtina 10000, Kosovo

**Keywords:** acute abdominal pain, cecal epiploic appendices, torsion

## Abstract

The epiploic appendix is rarely found in the cecum of children as a cause of abdominal pain in children due to torsion or inflammation. The purpose of this study was to reveal the preoperative diagnostic difficulties of cecal epiploic appendix torsion in children. We present the case of a 12-year-old girl who was misdiagnosed preoperatively with acute appendicitis and who was found upon surgical exploration to have a torsion of pedunculated tumor-like mass in the cecum. The uninflamed vermiform appendix and torqued mass-like tumor in the cecum were removed. The mass was confirmed to be a hemorrhagic infraction of the epiploic appendix of the cecum due to torsion. Pediatric surgeons should consider more often magnetic resonance image or eventually computed tomography of the abdomen and pelvis as the best diagnostic tool for cecal epiploic appendix torsion, especially when ultrasound reveals a non-inflamed vermiform appendix to avoid unnecessary surgical exploration.

## Introduction

Epiploic appendices of the colon are fatty structures covered with visceral peritoneum, mainly they are pedunculated, which predisposes them to torsion causing abdominal pain. In 1908, Briggs first reported a case of epiploic appendix torsion mimicking appendicitis [1]. Epiploic appendagitis, usually caused by torsion or inflammation of the epiploic appendix. Only a few reports of cecal epiploic appendagitis involving young children have been published [2, 3]. Although preoperative diagnosis is very difficult because of its rarity and similarity with other pathologies that cause right lower quadrant (RLQ) related abdominal pain, the recognition of this pathology by pediatric surgeons to avoid unnecessary surgical intervention. Preoperatively can be diagnosed mainly with magnetic resonance image or computed tomography (CT). Torsion or inflammation of the epiploic appendices in the cecum is rarely found to cause acute abdominal pain in RLQ in children and is usually mistaken for acute appendicitis.

## Case history

A 12-year-old girl Kosovo Albanian nationality was referred to our pediatric surgery department because of acute-onset abdominal pain in the RLQ that had begun a night before admission associated with nausea. Localized abdominal guarding and localized rebound tenderness in the right iliac fossa were noted. White blood cell count was 12.7 × 103/mm^3^. Other blood and urine tests were normal. Abdominal ultrasonography could not visualize the vermiform appendix but revealed minimal interloop free fluid. A presumptive diagnosis of acute appendicitis was made.

**Figure 1:**
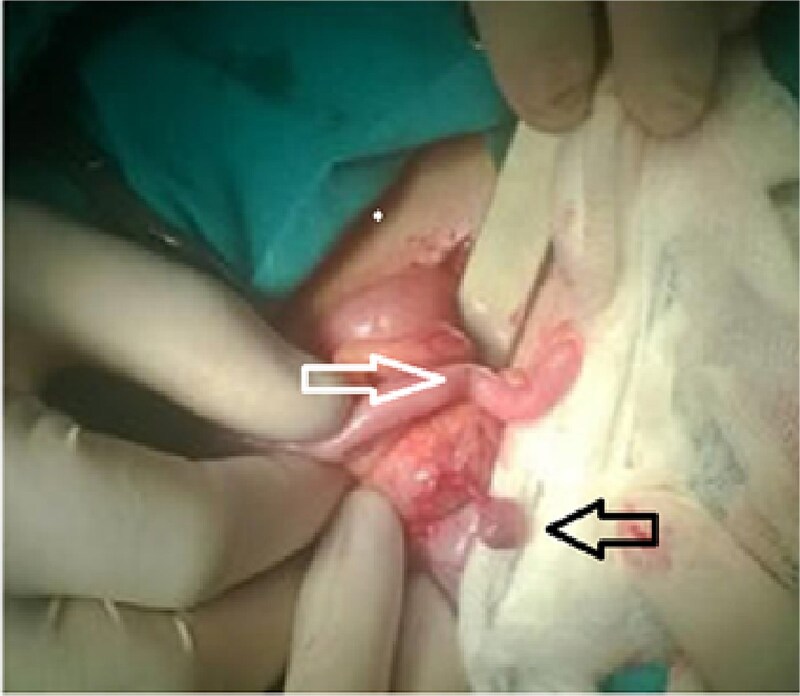
Torsion of the epiploic appendix of the cecum (black arow) with a normal appendix vermiform (white arow).

**Figure 2:**
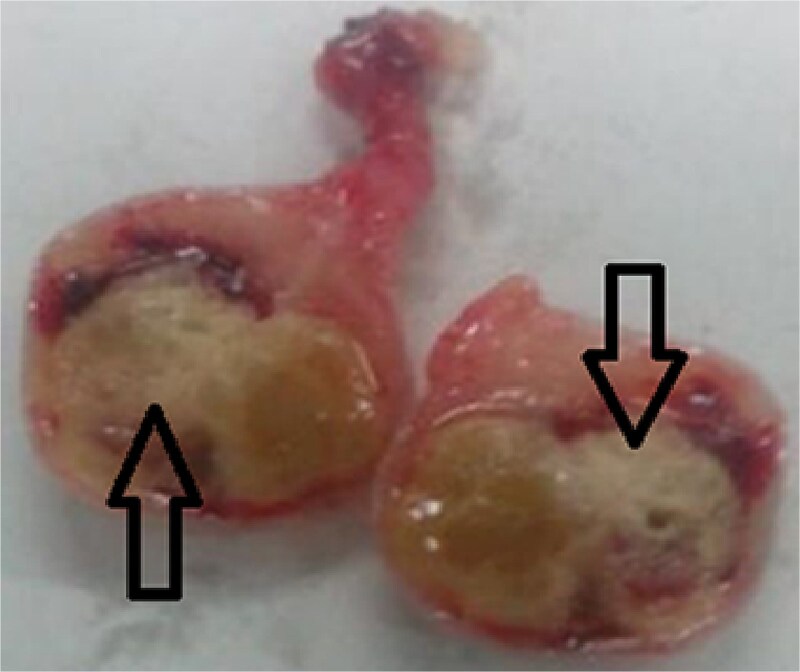
Ischemic infarct of the epiploic appendix adipose tissue (arrows).

The patient underwent surgery on an emergency basis. The appendix was not inflamed but near the appendix, a twisted epiploic appendix was observed (Fig. 1). Appendectomy and complete removal of the epiploic appendix were performed. The histological examination of the torqued mass revealed an ischemic infarct of adipose tissue in the twisted epiploic appendix (Fig. 2).

## Discussion

Epiploic appendices are small pedunculated fatty pouches on the colons of the antimesenteric surface composed of adipose tissue and blood vessels arising from the serosa of the colon surrounded by the peritoneum. Their peduncle shape and mobility make them prone to spontaneous torsion [4]. Although acute appendicitis is the main cause of acute abdominal pain in children, torsion of the epiploic appendix in children is rarely considered among pediatric surgeons. Only a few case reports of epiploic appendagitis of the cecum in children have been published in the literature [2, 3, 5]. With a diagnosis based on clinical manifestations alone, torsion of the cecal epiploic appendix is misdiagnosed in the majority of patients. In a series of 1320 patients with acute abdominal pain reported by Golash *et al*., only eight patients had acute epiploic appendagitis [6]. The preoperative diagnosis could be suspected in patients who had clinical signs and symptoms of acute appendicitis and a history of appendectomy [7]. Torsion of the cecal epiploic appendix is rarely diagnosed preoperatively due to the lack of nonspecific pathognomonic clinical features. Most patients manifest with acute-onset localized abdominal pain. Acute epiploic appendagitis involving the sigmoid colon can mimic diverticulitis of the sigmoid colon. If the proximal part of the transverse colon is involved, it may mimic acute cholecystitis [8]. In the cecum, it may mimic acute appendicitis or any other cause of acute pain in the right lower abdomen, such as regional enteritis, ovarian torsion or a ruptured ovarian cyst, salpingo-ophoritis, typhlitis, or peri typhlitis [9, 10]. The condition is self-limited, and the symptoms usually resolve within 1 week (mean of 4.7 days) without surgical treatment [2, 11]. Conservative treatment with nonsteroidal anti-inflammatory medication is sufficient. Recognition of these conditions on CT images will prevent unnecessary hospital admission, antibiotic therapy, laboratory tests and unnecessary surgery. Typically, epiploic appendages are visible on CT images when they are inflamed and/or surrounded by fluid as an oval lesion less than 5 cm in diameter with slightly greater attenuation than peritoneal fat connected to the serosal surface on the antimesenteric side of the colon [12]. But we should consider also the risk factor as an important point regarding ionizing radiation exposure associated with CT imaging in children. This imposes the limitation and the need to balance the diagnostic utility of CT against the potential long-term risks. We highlight that although CT can be highly informative in identifying epiploic appendagitis, its use in pediatric practice should be judicious, and alternative modalities such as ultrasound or magnetic resonance imaging (MRI) may be preferred when clinically appropriate. The detection of a solid, well-delineated hyperechoic mass at the site of maximum tenderness adjacent to the colonic wall by ultrasound and the absence or lack of blood flow observed on color Doppler images may suggest a diagnosis [13]. In our case, abdominal ultrasonography could not visualize the vermiform appendix despite being non-inflamed. The operator-dependent nature of pediatric ultrasound, the possibility of obscuring bowel gas, and the influence of patient body habitus plays a great role on visualization of appendicitis, epiploic appendagitis, or torsion of epiploic appendage. Thus, we consider ultrasonography may not be the best diagnostic option for suspected cases of cecal epiploic appendix torsion. MRI findings include an ovoid mass with a central portion of the mass seen as hyperintense and peripheral as hypointense on T1- and T2-weighted images [2, 14]. Currently, laparoscopy has been found to be useful for both the diagnosis and treatment of cecal epiploic appendix torsion [15]. If the diagnosis is made upon exploration, it should be removed, and seromuscular inversion of the affected portion of the gut should be performed [3]. Pediatric surgeons should consider epiploic torsion as a potential cause of RLQ acute abdominal pain in children, especially in patients who have undergone appendectomy previously and also in those associated with a lack of laboratory and radiological features of acute appendicitis. Since it can be treated conservatively, when suspected, it should be confirmed with CT or MRI examination to avoid unnecessary surgical exploration.
